# Vav1 Promotes B-Cell Lymphoma Development

**DOI:** 10.3390/cells11060949

**Published:** 2022-03-10

**Authors:** Batel Shalom, Marganit Farago, Yaser Salaymeh, Shulamit Sebban, Eli Pikarsky, Shulamit Katzav

**Affiliations:** 1 Department of Developmental Biology and Cancer Research, Institute for Medical Research Israel-Canada, Hadassah Medical School, Hebrew University, P.O. Box 12272, Jerusalem 91120, Israel; batel.shalom@mail.huji.ac.il (B.S.); marganitf@ekmd.huji.ac.il (M.F.); yaser.salaymeh@mail.huji.ac.il (Y.S.); shushub@ekmd.huji.ac.il (S.S.); 2 Department of Immunology and Cancer Research and Department of Pathology, Institute for Medical Research Israel-Canada, Hadassah Medical School, Hebrew University, Jerusalem 91120, Israel; peli@hadassah.org.il

**Keywords:** Vav1, Rac-GTP, B-cell lymphoma, Rosa26

## Abstract

Vav1 is normally and exclusively expressed in the hematopoietic system where it functions as a specific GDP/GTP nucleotide exchange factor (GEF), firmly regulated by tyrosine phosphorylation. Mutations and overexpression of Vav1 in hematopoietic malignancies, and in human cancers of various histologic origins, are well documented. To reveal whether overexpression of Vav1 in different tissues suffices for promoting the development of malignant lesions, we expressed Vav1 in transgenic mice by using the ubiquitous ROSA26 promoter (Rosa Vav1). We detected Vav1 expression in epithelial tissues of various organs including pancreas, liver, and lung. While carcinomas did not develop in these organs, surprisingly, we noticed the development of B-cell lymphomas. Rac1-GTP levels did not change in tissues from Rosa Vav1 mice expressing the transgenic Vav1, while ERK phosphorylation increased in the lymphomas, suggesting that signaling pathways are evoked. One of the growth factors analyzed by us as a suspect candidate to mediate paracrine stimulation in the lymphocytes was CSF-1, which was highly expressed in the epithelial compartment of Rosa Vav1 mice. The expression of its specific receptor, CSF-1R, was found to be highly expressed in the B-cell lymphomas. Taken together, our results suggest a potential cross-talk between epithelial cells expressing Vav1, that secrete CSF-1, and the lymphocytes that express CSF-1R, thus leading to the generation of B-cell lymphomas. Our findings provide a novel mechanism by which Vav1 contributes to tumor propagation.

## 1. Introduction

Many deregulated signal transducer proteins that are involved in cancer contribute to the ability of cells to over proliferate and escape mechanisms that normally control their survival and migration. These alterations might also lead to cancer progression through changes in the tumor microenvironment, angiogenesis, and inflammation. Deciphering the involvement of such signaling proteins is of outmost importance to the understanding of cancer development. Our study is focused on the role of the signal transducer Vav1 in cancer of various histological origins.

Vav1 was identified as a transforming gene, due to loss of its amino-terminus in a nude mice tumorigenicity assay [1]. Wild-type Vav1 (herein Vav1) was subsequently recognized as a cardinal signal transducer protein in the hematopoietic system, where it is exclusively expressed [2,3,4]. Tyrosine phosphorylation regulates the activity of Vav1 functions as a specific GDP/GTP nucleotide exchange factor (GEF) [2,3,4,5]. Mutations in various domains of the Vav1 protein are present in human cancers such as adult T-cell leukemia/lymphoma [6], peripheral T-cell lymphomas [7], lung adenocarcinoma and squamous cell carcinomas [7], as well other cancers (https://cancer.sanger.ac.uk/cosmic/ (accessed on 15 December 2021)). In addition, numerous studies have reported the unexpected expression of Vav1 in a variety of human cancers of various histological origin including the hematopoietic system [7,8,9], as well as solid tumors such as neuroblastoma [10], lung [11], pancreas [12], breast [13,14], ovarian [15], prostate [16], esophageal [17], and brain tumors [18]. Overexpression of signal transducer proteins, such as EGFR, HER2, Ras, and others in human cancers was reported [19]. Thus, expression of a signal transducer protein in a non-physiological context might overwhelm the normal regulatory control of cellular proliferation. Vav1 can be activated by various receptors in human cancers when it is overexpressed in non-hematopoietic cells, leading to signaling cascades similar to those it regulates in hematopoietic cells, including cytoskeletal reorganization and transcription [20,21].

Despite the reports on the involvement of Vav1 in human cancer, either as a mutated gene or an abnormally expressed gene, little work has been conducted to decipher its involvement in the development of cancer by using genetically engineered mouse models (GEMMs). Our laboratory was the first to analyze whether wild-type Vav1 leads to tumor generation in vivo [22]. We generated a novel transgenic mouse model that inducibly expresses Vav1 and mutant K-Ras^G12D^ in the pancreas. We showed that co-expression of Vav1 and mutant K-Ras dramatically increases the prevalence and decreases the time course required for malignant pancreatic lesions to appear compared to results obtained in mice that express only K-Ras^G12D^. Our results undoubtedly indicated that Vav1 and mutant K-Ras proteins synergize to augment the development of pancreatic tumors [22]. Fukumoto K, et al., analyzed the effects of activating mutations in Vav1 in the development of T cell neoplasms in transgenic mice and established the association between Vav1 mutations and malignant transformation of T cells [23].

The question is whether the mere overexpression of Vav1 in certain cells of non-hematopoietic origin suffices to lead to tumor development. We approached this issue by using animal models. The expression of Vav1 alone in the pancreas did not generate any malignant lesions. To find out whether the sole expression of Vav1 in other tissues could lead to the development of malignant lesions, we sought to express Vav1 ubiquitously in transgenic mice by using the Reverse Orientation Splice Acceptor (ROSA)βgeo26 (ROSA26) promoter (RosaVav1) [24]. The resulting RosaVav1 mice overexpress transgenic Vav1 in multiple epithelial tissues, but we could not detect its expression in lymphoid tissues. Surprisingly, overexpressing Vav1 in epithelial tissues induced chronic inflammatory reactions eventually leading to B-cell lymphomas development. Our analysis indicated that ERK phosphorylation increased in the lymphomas, suggesting that signaling pathways are evoked. Finally, while the growth factor CSF-1 is highly expressed in the epithelial compartment, the expression of its specific receptor is noticeable in the lymphomas. Taken together, our results suggest that abnormal expression of wild-type Vav1 in certain histological compartment leads to changes in signal transduction pathways as well as in growth factor/cytokine expression, which might contribute to development of cancer in adjacent histological compartments. 

## 2. Materials and Methods

### 2.1. Mouse Strains

**Figure 1:**
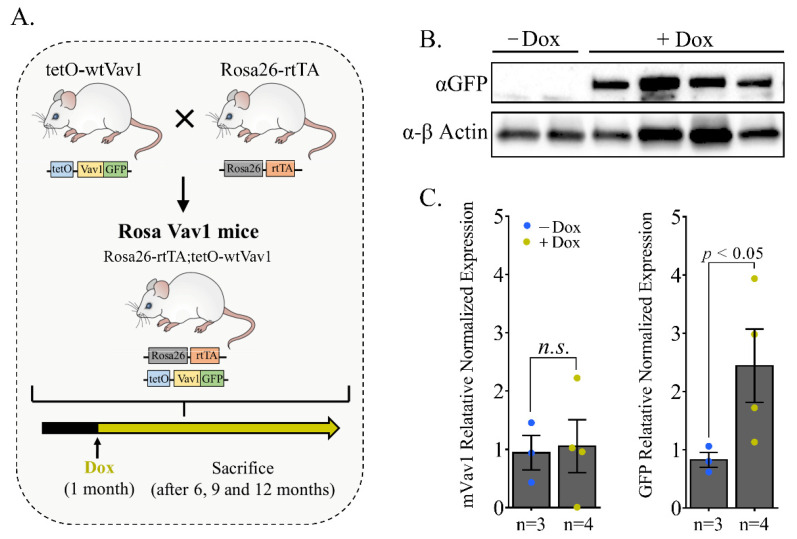
RosaVav1 transgenic mouse model (**A**) and expression pattern (**B**). (**A**) Schematic diagram of breeding strategy to generate RosaVav1 transgenic mice. Rosa26-rtTA mice were crossed with tetO-wtVav1 mice to generate Rosa26-rtTA/tetO-wtVav1 mice (Rosa Vav1 mice). To induce Vav1 expression, mice were treated with Dox one month after birth. Mice were sacrificed at different time points (6, 9, and 12 months after transgene induction). (**B**) Expression of transgenic Vav1 protein in the lungs of Rosa Vav1 mice. Western blotting using anti–GFP (indicative of the transgene humanVav1 expression) and anti-Actin Abs. (**C**) Expression of transgenic human Vav1 mRNA (GFP) and murine Vav1 (mVAV1) in the lungs of Rosa Vav1 mice. Quantitative real-time PCR analysis showing the mean mRNA expression of murine Vav1 (mVav1; left panel) and GFP (human transgenic Vav1; right panel) in lung tissues from Rosa Vav1 mice either treated (+Dox; *n* = 4) or non-treated (−Dox; *n* = 3), 12 months post transgene induction is depicted. SEM and significance between the treated (+Dox) and the non-treated (−Dox) analyzed by *t*-test are indicated. n.s. means non-significant.

Rosa Vav1 transgenic mice were generated by crossing Rosa26-rtTA mice (purchased from Jackson laboratories, Bar-Harbor, ME, USA) with tetO-wtVav1 mice, previously generated by Salaymeh et al. [22]. Briefly, human wild-type Vav1 was subcloned into a plasmid that encodes a Tet-O responsive bidirectional promoter (Teto7minCMV) driving expression of wild-type human Vav1 hooked to GFP (tetO-Vav1). TetO-Vav1 mice were produced by injecting the plasmid into C57BL/BALB/c blastocysts according to standard IVF protocol. TetO-Vav1 mice were crossed with Rosa26-rtTA mice to generate Rosa Vav1 mice (Figure 1A). To induce Vav1 expression in Rosa-Vav1 mice, Doxycycline (Dox, 0.5 mg/mL, Bio-Basic Inc., Markham, ON, Canada) was provided in the drinking water as a sucrose solution (3% *w*/*v*) to 1-month old mice. All experiments were approved by the Hebrew University Ethics Committee for animal use (#MD-19-15940-5).

### 2.2. Mice Genotyping

PCR analysis was performed on tissue lysates of either tail or ear. DNA was extracted using KAPA mouse genotyping kit (Kapa Biosystems) according to the manufacturer’s protocol. Detection of the human Vav1-GFP transgene was performed using specific primers (Supplementary Table S1A). Reactions were amplified as follows: 95 °C denaturation for 5 min, followed by 35 cycles of 95 °C for 30 s, 63 °C for 30 s, 72 °C for 30 s, followed by a 5 min extension at 72 °C. PCR products were resolved on a 1.5% agarose gel. 

### 2.3. Real Time 

Total RNA was isolated from the lung lobe, liver, and gut of Rosa Vav1 mice after Vav1 transgene induction, using TRI reagent (Sigma Kiryat HaMada, Jerusalem, Israel), and the miRNeasy Kit (QIAGEN, Maryland, MD, USA) according to the manufacturer’s instructions. cDNAs were prepared using the qScript reverse transcriptase (Quanta, Taipei, Taiwan). Quantitative PCR (qPCR) was performed by using the CFX-384 real-time PCR machine (Bio-Rad, Hercules, CA, USA) to detect Vav1 transgene, mVAV1, and GFP using cyber green PCR master mix (Bio-Rad, Hercules, CA, USA) and the required primers (Supplementary Table S1B). Three independent experiments were performed, and in each experiment, triplicates were used.

### 2.4. Western Blotting

Lung and liver tissues were fragmented using a homogenizer (Polytron–Kinematica) and lysed in the following lysis buffer: 50 mM Tris-HCL, 150 mM NaCl, 25 mM EDTA, 1% NP-40, 1× Phosphatase and Protease Inhibitors (Roche) and 1% PMSF- Phenylmethylsulphonyl Fluoride). Lysed tissues were incubated for 30 min on ice, then centrifuged for maximum speed for 20 min. Equal amounts of proteins were loaded on 4–15% gradient acrylamide gels. Antibodies used for western blotting are listed in the supplementary materials (Supplementary Table S2). Quantification of the western blot results was performed using ImageJ 1.49 V1 (NIH, Bethesda, MD, USA). 

### 2.5. Analysis of GEF Activity of Vav1

Rac1 activation of Vav1 in tissues of Rosa Vav1 mice were determined by western blotting using anti-Rac1-GTP mAbs (Supplementary Table S2) or by GST-PAK pull-down assay as described by Lazer et al. [11].

### 2.6. Histology, Immunohistochemical and Immunofluorescence Analysis

Paraffin-embedded serial sections of pancreas, lung, and liver at different time points after induction of Rosa Vav1 mice were stained with Hematoxylin and Eosin (H&E) and histological analysis was performed using standard procedures. Immunohistochemical or immunofluorescence staining were performed as described (https://www.abcam.com/kits/introduction-to-immunohistochemistry-ihc (accessed on 18 December 2021)). Detailed list of primary and secondary antibodies can be found in the supplementary materials (Supplementary Table S2). Staining was evaluated by a board-certified pathologist (E.P.).

### 2.7. Histological Analysis

H&E-stained sections of lung, liver and pancreas from the Rosa Vav1 mice were quantified for malignant lesions. Histopathological evaluation was carried out by calculation of percentage area of tumor as a fraction of the total section using analysis software (Image J, 1.49 V1, NIH, Bethesda, MD, USA) through the following equation: % of tumor area  =  (area of tumor/total area of the field) × 100. Also, the total tumor numbers for each group were determined by analyzing H&E-stained sections.

### 2.8. Statistical Analysis

*p*-values were calculated using the two-tailed student’s *t*-test with the GraphPad Prism or Excel software. *p* < 0.05 indicates a significant difference. 

## 3. Results

### 3.1. Generation of a Rosa26 Inducible Vav1 Transgenic Mouse 

To determine whether unbiased expression of wild-type Vav1 (Vav1) in adult mouse tissues leads to the development of cancer, we generated a Vav1 transgenic mouse line that is driven by the Rosa26 promoter. Rosa26 is a locus used for constitutive, ubiquitous gene expression in mice [24]. Over 130 knock-in lines have been created based on the Rosa26 locus [25]. We used the Tet-On system, in which rtTA binds, only in the presence of Doxycycline (Dox), to a tetO plasmid. Thus, as detailed in Salaymeh et al. [22], we subcloned human Vav1 into a plasmid that encodes a tetO-responsive bidirectional promoter (tetO7minCMV) that drives expression of tetO-Vav1 hooked to GFP. These mice were crossed to Rosa26–rtTA transgenic mice to generate Rosa26-rtTA;tetO-Vav1, herein Rosa Vav1 transgenic mice (Figure 1A). 

To validate the expression of GFP only upon Dox treatment, we extracted proteins and RNA from tissues of Rosa Vav1 transgenic mice either treated (+Dox) or non-treated (-Dox). RT-PCR of mRNA extracted from various tissues a month after transgene induction showed that the Vav1 transgene is found in the liver, lung, and gut at various levels, while no expression was detected in non-treated mice (Supplementary Figure S1A). These results were further verified by western blotting with anti-Vav1 antibodies (Supplementary Figure S1B). We then evaluated the expression of the human Vav1 transgene in lungs of mice 12-months post-transgene induction (Figure 1B,C). Analysis of GFP protein expression, as a marker for the expression of the human Vav1 transgene indicated that following treatment with Dox, the expression of GFP protein was observed in lungs (Figure 1B), thus further highlighting the expression of GFP in the lungs of mice treated with Dox as opposed to no expression in the lungs of mice that were not treated. RT-PCR of mRNA extracted from lungs of RosaVav1 mice, indicated that while there was no difference in the expression of mouse Vav1 in the lungs with or without Dox treatment as expected (Figure 1C, left panel), significant differences are observed in the expression of human Vav1 transgene between the treated (+Dox) and non-treated mice (−Dox, Figure 1C, right panel). Thus, our results clearly demonstrate the dox dependent expression of the Vav1 transgene in various organs of mice treated with Dox.

### 3.2. Malignant Lesions in Rosa/Vav1 Mice

**Figure 2:**
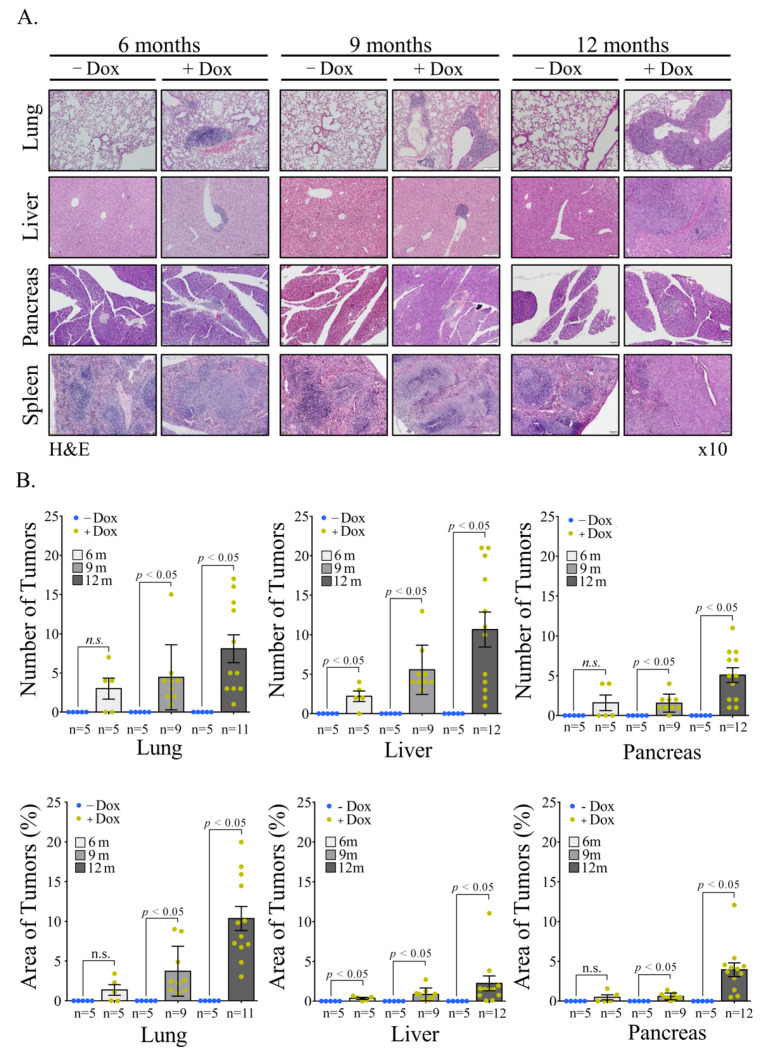
Appearance of Malignant lesions in Rosa Vav1 mice. (**A**) Representative images of hematoxylin and eosin (H&E) staining of lung, liver, pancreas, and spleen sections from Rosa Vav1 mice either treated (+Dox) or non-treated (−Dox), at indicated time points after transgene induction. Magnification at ×10. (**B**) Quantitative analysis of tumor numbers (upper panel) and percentage area (lower panel) in H&E-stained sections. The quantitation of area of tumors is detailed in the Material section. Number of mice used: non-treated (−Dox; 6, 9, 12 months *n* = 5); treated (+Dox; 6 months *n* = 5; 9 months *n* = 9; 12 months *n* = 12). SEM and significance between the treated (+Dox) and the non-treated (−Dox) at each time point analyzed by *t*-test are indicated.

We then analyzed various organs of Dox-treated Rosa Vav1 mice for the appearance of malignant lesions. Mice were euthanized at various time points (6, 9, and 12 month) after transgene induction was initiated by Dox administration, and various tissues were removed and analyzed by hematoxylin and eosin (H&E) staining for the appearance of malignant lesions, as presented in Figure 2A. Appearance of lesions in the pancreas, liver, lung, and spleen were observed already at six months after transgene induction and onwards. Representative examples of sections from these tissues are depicted in Figure 2A. An expert pathologist categorized the lesions as lymphomas based on cellular morphology and the ability of the lesions to destruct tissue architecture. For each section of the lungs, livers and pancreas, we calculated the number of lymphomas and the area that they occupy compared to the total area of the section analyzed as detailed in the Materials and Methods Section (tumor area, Figure 2B). The number of tumors increased with time and was statistically significant compared to tissues of mice that are not treated with Dox (−Dox). The area that the lymphomas occupy was the highest in the lungs (Figure 2B). Yet, it is important to note that it was impossible to count the number of lymphomas in the spleen as most of the organ was occupied. We did, however, weighed and measured the lengths of spleens from Dox-treated and non-treated RosaVav1 mice (Supplementary Figure S2). The results clearly indicate an increase in both parameters analyzed, indicative of the development of lymphomas in this organ. Thus, an induction of lymphomas is observed in Rosa Vav1 mice once Vav1′s expression is induced by Dox treatment.

### 3.3. Characterization of Lymphomas in Rosa26/Vav1 Transgenic Mice

**Figure 3:**
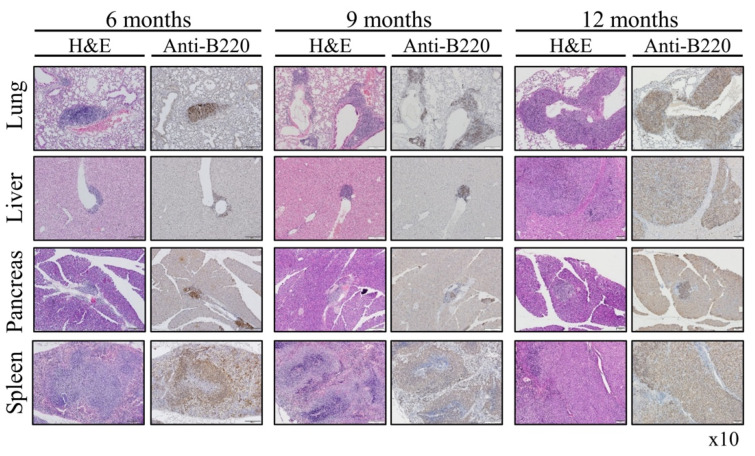
Presence of B cells in the Lymphomas of Rosa Vav1 mice. Sections of lung, liver, pancreas, and spleen from Rosa Vav1 mice either treated (+Dox) or non-treated (−Dox) at indicated time points after transgene induction, were stained with H&E and anti-B220 antibodies. Representative pictures are shown. Magnification at ×10.

We then characterized the type of lymphoma that appeared in Rosa Vav1 mice. For that, we stained the various sections with anti-B220 Abs (Figure 3). B220 is a 220 kDa transmembrane protein tyrosine phosphatase, also known as CD45R, and is commonly used as a B cell marker [26]. Immunohistochemistry (IHC) staining of sections from the lung, liver, pancreas, and spleen, removed at different time points following Dox administration, with the Anti-B220 Abs revealed that the malignant lymphoma cells were of B cell origin. Immunostaining with anti-CD3 highlighted normal appearing reactive small T cells that were dispersed in the lesions in line with a classical morphology of low-grade B-cell lymphoma in humans (Supplementary Figure S3). 

**Figure 4:**
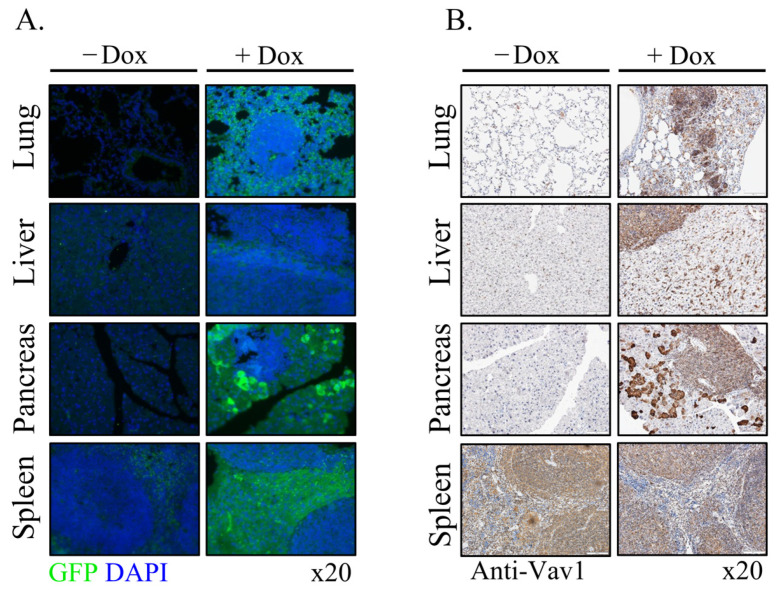
Expression of transgenic human Vav1(indicated by GFP) in the epithelial tissue at various organs of Rosa Vav1 mice. Sections of lung, liver, pancreas, and spleen from Rosa Vav1 mice either treated (+Dox) or non-treated (−Dox), Dox 12 months post transgene induction were stained with anti-GFP antibodies that identify the human Vav1 transgene (immunofluorescence; green) (**A**) or anti-Vav1 antibodies that identify murine and human Vav1 (**B**). Representative pictures are shown. Magnification at ×20.

Since Vav1 is physiologically expressed in the hematopoietic system, it was expected that the development of B-cell lymphomas in the different organs stems from the extra expression of Vav1 in this system due to the contribution of the transgenic human Vav1. To further explore this possibility, we stained sections prepared from the lung, liver, pancreas, and spleen for GFP which is co-expressed with the human Vav1 transgene (Figure 4A). Surprisingly, transgenic Vav1 as visualized by GFP staining was noted mainly in the epithelial compartment of each of the various organs. Note that the lung, pancreas and spleen sections in Figure 4A also harbor lymphoma lesions, and these do not express GFP at all. We also stained the tissue sections with anti-Vav1 Abs that stains both human and murine Vav1 (Figure 4B). As visualized by the IHC staining, Vav1 is found in the lymphomas as expected (likely representing the endogenous Vav1), but it is also expressed in epithelial cells, confirming the results obtained with the GFP staining (Figure 4A). Of note, expression of Vav1 is also observed in non-treated RosaVav1 mice, indicative of its normal expression in this organ. This conclusive result raises the possibility that the mere abnormal expression of the transgenic Vav1 in the epithelial tissue of an organ contributes to the generation of lymphomas. 

### 3.4. Signaling Pathways in Rosa Vav1 Mice

**Figure 5:**
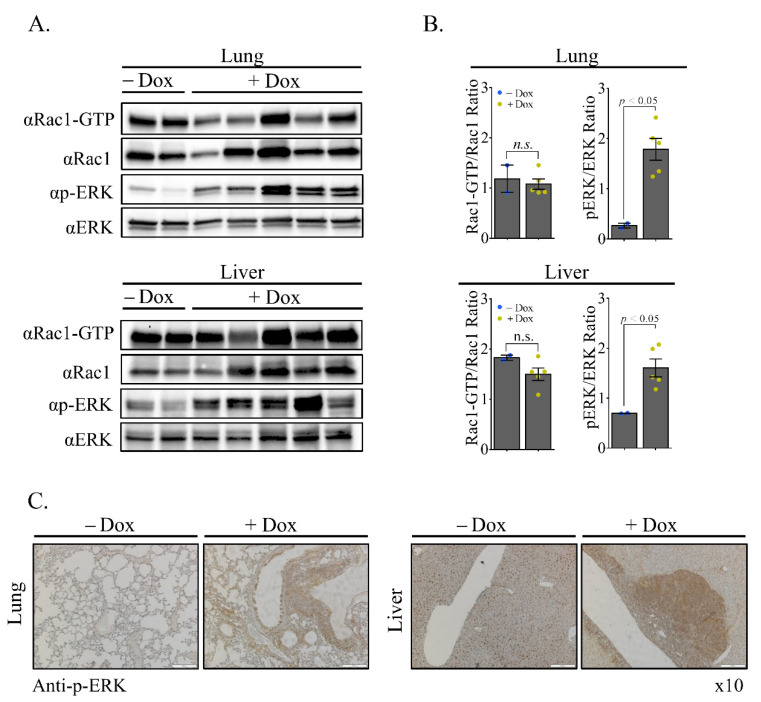
Activation of ERK in Lymphomas from Rosa Vav1 mice. Analysis of Rac1 activation in the lung and ERK activation in the lung and liver of Rosa Vav1 mice either treated (+Dox) or non-treated (−Dox), 12 months post transgene induction was performed. (**A**) Protein lysates from _Rosa Vav1 transgenic mice were evaluated Rac1-GTP activation (upper panel; lung and liver tissues) and ERK phosphorylation (lower panels; lung and liver respectively) by using anti-Rac1-GTP, anti-Rac1, anti-pERK and anti-ERK Abs in western blotting. (**B**) The relative ratios of Rac1-GTP/Rac1 and pERK/ERK were calculated from the blots shown in A. The mean intensity of the western blots was quantified using ImageJ 1.49 V software. Numbers of mice used: non-treated (−Dox; *n* = 2); treated (+Dox; *n* = 5). SEM and significance between the treated (+Dox) and the non-treated (−Dox) analyzed by *t*-test are indicated. (**C**) Sections of lung (left) and liver (right) from indicated mice were stained with anti-pERK antibodies. Representative pictures are shown. Magnification at ×10.

Vav1 is a well-known signal transducer that can affect tumor microenvironment through its involvement in numerous signaling pathways. The best-known function of Vav1 is its tyrosine phosphorylation–dependent GEF activity for the Rho family of GTPases [5]. Several studies indicated that the activity of Vav1 as a GEF towards Rac1 plays an important role in Vav1’s involvement in cancer [11,12]. Therefore, we wished to determine whether activation of Rac1 might play an indirect role in the contribution of Vav1 to development of Lymphomas. This approach was used by us previously by Salaymeh et al., who showed that Rac1-GTP levels in in the pancreas of transgenic K-Ras^G12D^/Vav1 mice is significantly higher compared to its level in the pancreas of other transgenic mice used in the study [22]. We analyzed Rac1 activation using specific anti–Rac1-GTP Abs in the lung in which lymphomas developed (Figure 5A, upper panel). Surprisingly, there were no differences in Rac1 activity in the lung tissue from Rosa Vav1 mice either treated or non-treated with Dox, although Rac-GTP activity was recorded (Figure 5A). Quantification of the western blot further substantiates this conclusion (Figure 5B). Furthermore, a pull-down with GST-PAK further strengthens the conclusion of lack of Rac1 activation in Rosa Vav1 mice treated with Dox (Supplementary Figure S4).

Tyrosine phosphorylation of Vav1 following stimulation of various receptors leads to its activation, which triggers downstream signaling cascades, including ERK [27,28,29,30]. The potential link between Vav1 activity in ERK phosphorylation was noted in hematopoietic cells, where Vav1 is physiologically active, as well as in cancer cells. Therefore, we examined whether the expression of transgenic Vav1 protein affects ERK activation in the organs that develop lymphomas. For that, we produced protein extracts from lung and liver (Figure 5) and analyzed them by western blotting for the status of ERK phosphorylation (Figure 5A,B). ERK phosphorylation was increased substantially in the lungs and livers of Rosa Vav1 mice treated with Dox compared to its status in control tissues prepared from non-treated mice (Figure 5A, lower panels). Thus, a clear increase in ERK phosphorylation is noted in western blotting of lung and liver tissues (Figure 5A). These results were further substantiated by quantifications (Figure 5B). 

**Figure 6:**
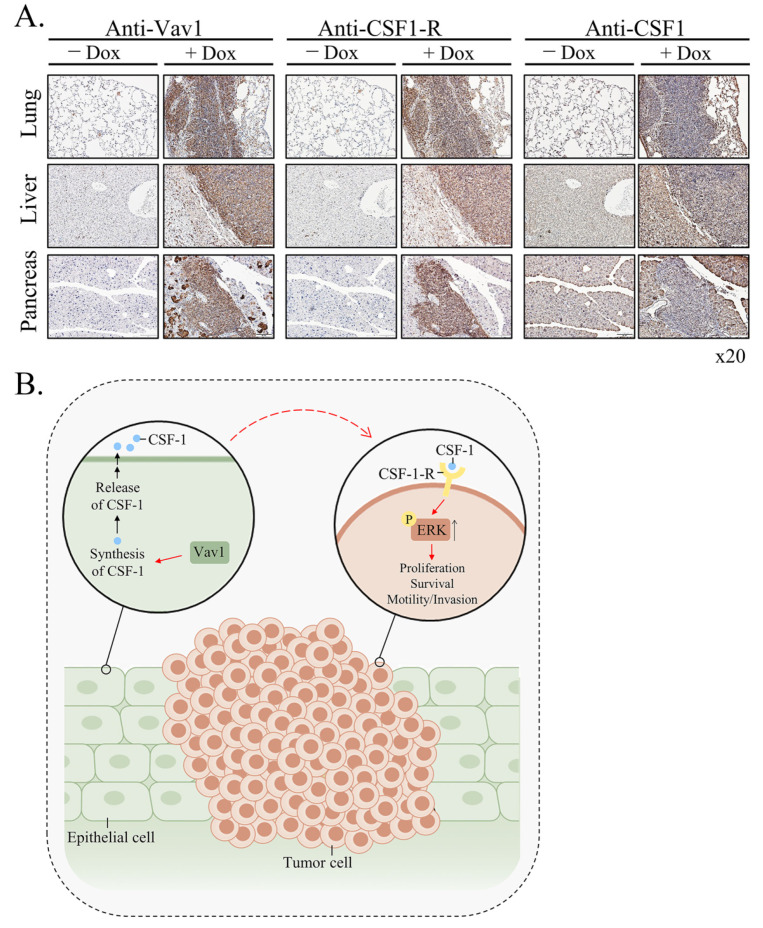
Mechanism of generation of B-cell Lymphomas in Rosa Vav1 mice. (**A**) Sections of lung, liver, and pancreas from Rosa Vav1 mice either treated (+Dox) or non-treated (−Dox), 12 months post transgene induction stained with anti-Vav1, anti-CSF-1R and anti-CSF1 antibodies are depicted. Representative pictures are shown. Magnification at ×20. (**B**) The model proposed for generation of B-cell lymphomas from due to the aberrant expression of Rosa Vav1 in epithelial cells suggests that CSF-1 secretion from the epithelial compartment activates the CSF-1R on B-cells, leading eventually to the development of B-cell lymphoma.

Next, we sought out to identify the specific tissue compartments in which ERK is activated. IHC staining with anti-phospho ERK Abs revealed that the increase was mostly noted in lymphomas and chronic inflammatory infiltrates present in the lung and liver (Figure 5C). These results raised the possibility that the expression of transgenic-Vav1 instigated ERK signaling in the lymphoma cells. We previously showed that the expression of Vav1 in epithelial lung cancer cells is associated with the expression of colony-stimulating-factor-1 (CSF1), a hematopoietic growth factor [30]. Thus, we explored whether CSF-1 and its receptor are expressed in the epithelial or the lymphoma tissue compartments. As demonstrated by IHC of lung, liver and pancreas sections with anti-Vav1, anti-CSF-1R and anti-CSF-1 Abs, while CSF-1R is expressed in cells within the B-cell lymphoma, the ligand is expressed in the epithelial tissue where transgenic Vav1 is found. These results raise the possibility that Vav1 overexpression in epithelial cells leads to synthesis and secretion of CSF-1, which in turn may activate CSF1-R in lymphoma cells, thus possibly promoting B cell lymphoma development (Figure 6B).

## 4. Discussion

The overexpression of Vav1 in hematopoietic malignancies [8,9], and in human cancers of varied histologic origins is well documented [10,11,12,13,14,15,16,17,18]. Prieto-Sanchez et al. [8], reported overexpression of Vav1 in 10 of 14 cases of B-CLL with 13q deletion. Hollmann et al. [31] demonstrated a link between the expression of Vav1 and CD40-mediated apoptosis in diffuse large B-cell lymphoma cell lines. Vav1 expression was linked to sensitivity/resistance to CD40 stimulation between DLBCL cell lines. In a malignant T-cell line, Yin, et al. [32] have established an association between increased Vav1 expression and increased Bcl2 expression which is associated with decreased sensitivity to fas-mediated apoptosis, but to our knowledge, this has not been described in malignant B-cell. Vav1 was shown to be required ATRA-induced differentiation in acute myeloid leukemia (AML) cell lines to neutrophils and to maturation of these same cell lines to monocytes/macrophages following PMA treatment [33,34]. Vav1’s overexpression in epithelial tissues where it leads to tumor generation was thus far attributed to its promoter methylation status. In human lymphocytes, Vav1’s promoter is unmethylated, but it is methylated to various degrees in cancers of non-hematopoietic tissues, that do not normally express Vav1 [12,18,35]. Another mechanism pointed to the involvement of changes in putative transcription factor binding sites at the Vav1 promoter that affect its transcription in cells of various histological origins [36].

The potential contribution of Vav1 to oncogenesis was mainly investigated by IHC analysis of human specimens [11,12,13,15,16,17,20] and by the use of cell lines [1,37,38]. These studies provided invaluable insights into the association between Vav1 expression and prognosis in multiple tumor types. Our experiments with transgenic mice expressing Vav1 in the pancreas demonstrated that the mere expression of Vav1 in this organ does not lead to tumor formation. To find out whether this is a general phenomenon, we expressed Vav1 under a ubiquitous promoter, Rosa26, and followed tumor generation. Although, the ROSA26 locus is widely used as a locus for expressing transgene sequences, our results indicate that it mainly drives the Vav1 transgene’s expression in the epithelial tissue, and not in the lymphoid tissues. It is possible that such an event is the result of transcriptional interference with the endogenous ROSA26 promoter, as was shown to take place by Strathdee et al. [39,40]. The same phenomenon was demonstrated for the β-globin locus control region that can silence as well as activate gene expression [40]. We show here, for the first time, that expression of Vav1 in the epithelial tissue compartment leads to generation of B-cell lymphomas in various organs such as lungs, liver, pancreas and spleen (Figure 2, Figure 3 and Figure 4).

Host defense, inflammation, organogenesis, tissue repair, cancer growth, and immunity are all regulated by a complex network of epithelial cells and leukocytes. Bidirectional interactions, rather than the functions of individual cell types, contribute to tissue integrity and immunological homeostasis under steady-state conditions, while they can produce a complex pathologic tissue microenvironment leading to disease development. Indeed, the question then arises as to is how can the expression of Vav1 in one histologic compartment, the epithelium, lead to development of tumors of a different histologic origin, B-lymphomas. One possible example is that of Mucosa-associated lymphoid tissue (MALT) lymphomas that originate in sites of chronic epithelial inflammation in various sites of the body, where it plays a role in regulating mucosal immunity [41]. MALT lymphomas are present in the gastrointestinal tract [42], nasopharynx [43], thyroid [44], breast [45], lung [46], salivary glands [47], eye [48], and skin [49] and exhibit characteristics with B cells located in the marginal zone of lymph node follicles. MALT lymphomas originate in sites of chronic epithelial inflammation. One example are MALT lymphomas in the stomach which are commonly caused by *Helicobacter pylori* infection [42]. The association between epithelial cells and lymphoma generated is best explored in patients with primary Sjögren’s syndrome (pSS), which is characterized by chronic hyperactivation of B lymphocytes, that exhibit an increased risk of development of non-Hodgkin lymphoma [42]. Salivary gland epithelial cells (SGECs) were shown to play a role in promoting B cell activation, differentiation and survival through direct interaction and cytokine production [42,47,50]. These cytokines include IL-6, B-cell activating factor (BAFF), and type I interferon leading to B cell activation, homeostasis, and survival [51,52,53,54,55,56]. SGECs were shown to express immune-competent molecules that regulate lymphocyte recruitment, homing, activation, differentiation and survival [57]. Thus, the crosstalk between SGECs and B cells suggests that salivary gland epithelial cells play a critical role in SS pathogenesis. The activation of Vav1 by various pathogens, such as Helicobacter pylori [58] and mycoplasma [59], that may drive MALT carcinogenesis was also suggested, yet it is not clear whether the B-cell lymphomas developed in Rosa Vav1 mice fit the same pathological classification.

One of the likeliest possibilities that B-cell lymphoma develop in Rosa Vav1-transgenic mice is that Vav1-epithelial expressing cells secrete ligands that affect B-cell proliferation. Indeed, our results clearly demonstrate the increased expression of CSF-1 in epithelial cells, while the expression of its receptor is found on the lymphoma cells (Figure 6). Vav1 was shown to be involved in increased secretion of ligands which function in an autocrine or paracrine fashion. Thus, the human mammary epithelial cell line MCF-10A, that ectopically expresses an oncogenic form of Vav1, exhibit increased migration and morphological changes, accompanied by secretion of an autocrine EGF receptor ligand [60]. Also, diminished Vav1′s expression in lung cancer cell lines reduced the expression of the growth factors, TGFα and EGF [60,61], and CSF-1 [30], which led to reduced tumorigenicity. Depletion of CSF1 in lung cancer cells led to decreased proliferation and focus-formation in vitro, as well as diminished tumor growth in immune-compromised mice, suggesting that CSF-1 secretion is cardinal for tumorigenicity [30]. Immunohistochemical analysis of Vav1 and CSF-1 expression of primary human lung tumors pointed to a strong link between these proteins, associated with to tumor grade. We also demonstrated that conditioned media from lung cancer cells contain a growth factor, potentially CSF-1, that activates U937 monocytic cells, and that conditioned media from U937 cells instigates signaling in lung cancer cells, thus suggesting a cross-talk mechanism between immune cells and lung cancer cells mediated by CSF-1. Thus, Vav1 could influence tumor growth and the tumor’s microenvironment via CSF-1 in an autocrine/paracrine mechanism. Such a potential mechanism was also shown for breast and ovarian carcinomas. Thus, the rate of tumor progression is reduced substantially in CSF-1-knockout mice [62]. Macrophages expressing EGF promote migration and invasiveness of breast carcinoma cells as well as CSF-1 expression by the latter, and cancer cell-derived CSF-1 is able to induce EGF production in macrophages [63]. Thus, cytokines/growth factors such as CSF-1 can mediate the interaction between epithelial tumor cells and inflammatory cells.

CSF-1 contributes to the survival, proliferation, and differentiation of mononuclear phagocytes and the female’s reproductive tract [64]. Under physiological conditions, CSF-1 is produced by fibroblasts, endothelial cells, monocytes, macrophages, osteoblasts, microglia, keratinocytes, bone marrow stromal cells, natural killer cells, B-cells and T-cells and epithelial cells [65,66]. It is an essential regulator of development and homeostasis of the mononuclear phagocyte system and, by extension, a key factor of CSF1-dependent macrophage control of development and homeostasis [64,67,68]. CSF-1 appears to play an autocrine and/or paracrine role in cancers of the ovary, endometrium, breast, lung, the nervous system, and myeloid and lymphoid tissues, within which overexpression of CSF-1 receptor is considered as a prognostic factor for survival in cancer [68].

Our results demonstrating the presence of CSF-1 in the epithelia of the various organs of Rosa Vav1 transgenic mice that develop B-cell lymphoma, points to the possibility that it can be produced in cells other than hematopoietic system, once signaling driven by proteins such as Vav1 are abnormally expressed (Figure 6). In the hematopoietic system, CSF-1 exerts its pleiotropic effects by binding to a single class of high-affinity receptors (CSF-1R) expressed predominantly on monocytes, macrophages, and their committed BM precursors [67]. Based on this knowledge, we expected the CSF-1 receptor to be expressed on monocytes within the B-Cell lymphomas identified in the Rosa Vav1 transgenic mice. However, no staining was detected when we used F4/80 antibodies that usually bind to macrophages from different sites including the peritoneal cavity, lung, spleen, and thymus, to blood monocytes and to macrophages derived from bone marrow precursors in culture (data not shown) [69], thus suggesting that the marked expression of CSF-1 receptor noted by us is expressed on other cells (Figure 6). Although CSF-1R expression is generally thought to be restricted to myeloid lineage cells, recent studies convincingly demonstrated its aberrant expression on non-myeloid lineage cells, including malignant B cells and classic Hodgkin lymphoma [70,71,72,73,74].

The emerging model from our studies depicted in Figure 6B is that aberrant expression of Rosa Vav1 in epithelial cells leads to CSF-1 secretion (Figure 6A). CSF-1 in turn activates the CSF-1R on B-cells and leads to enhancement of ERK phosphorylation (Figure 5) and cell propagation, leading eventually to the development of B-cell lymphoma. The fact that the mere expression of Vav1 in epithelial cells does not lead to the development of carcinomas further substantiates our previous studies with Vav1-pancreatic transgenic mice [22]. One would have expected, based on our current results, that Vav1 will lead to the generation of B-Cell lymphomas when expressed in the pancreas, but it’s expression in both studies was under different promoters, rosa26 in this study versus Ptf1a promoter in the pancreas, which might affect its expression and influence. Yet, it is obvious that Vav1’s ectopic expression impacts tumor development either in the pancreas when it is co-expressed with mutant K-Ras or when it is expressed in various tissues under the Rosa promoter.

## Data Availability

Not applicable.

## Supplementary Materials

The following supporting information can be downloaded at: https://www.mdpi.com/article/10.3390/cells11060949/s1, Figure S1: Expression of Vav1 in Rosa Vav1 mice; Figure S2: Analysis of spleens from RosaVav1 mice; Figure S3: Presence of T cells in the Lymphomas of Rosa Vav1 mice; Figure S4: Vav1 activity as GEF for Rac in liver tissue from Rosa Vav1 mice; Table S1: Primers used in this study; Table S2; List of antibodies used in this study.
