# Supplementary material for: Vav1 Promotes B-Cell Lymphoma Development
Source: Cells. 2022 Mar 10;11(6):949. doi: 10.3390/cells11060949 (PMC8946024; doi:10.3390/cells11060949)
Supplement: Supplementary file 1 [file cells-11-00949-s001.zip › cells-1562679-supplementary.pdf]

Supplementary Information

Supplementary Figure S1. Expression of Vav1 in Rosa Vav1 mice. A. Real-time PCR on mRNA from the indicated organs from Rosa26rtTA/tetO-wtVav1 mice either untreated (-) or one-month treated with doxycycline (+) was performed by using specific primers which span the human Vav1-GFP transgene. B. Lysates of lungs, liver, intestines from one-month doxycycline treated Rosa Vav1 mice (+/+) and their littermates negative for Vav1 transgene (-/-) immunoblotted with antibodies against Vav1 that detect endogenous murine Vav1 and the human Vav-transgene.

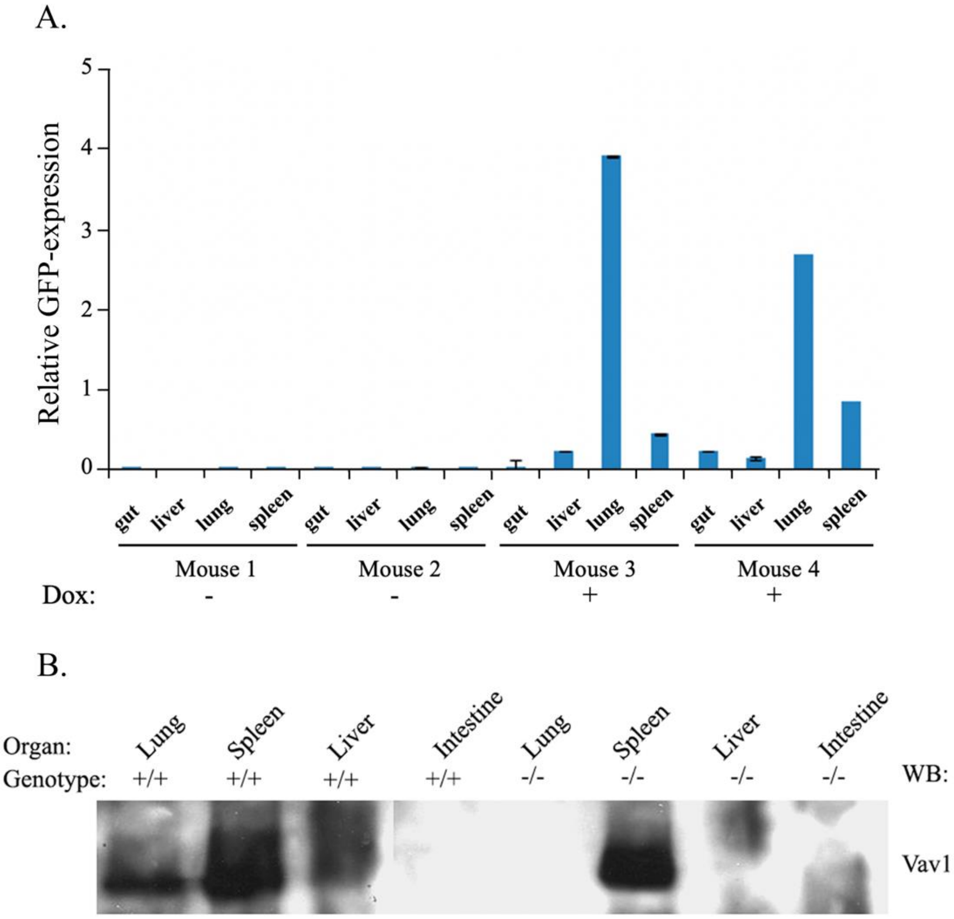

Supplementary Figure S2. Analysis of spleens from RosaVav1 mice. Quantitative analysis of spleen weight (left panel) and length (right panel) from RosaVav1 mice either treated (+Dox) or un-treated (-Dox) mice is depicted. Number of mice used is indicated below each column. SEM and significance between the treated (+Dox) and the untreated (-Dox) mice analyzed by t-test are indicated.

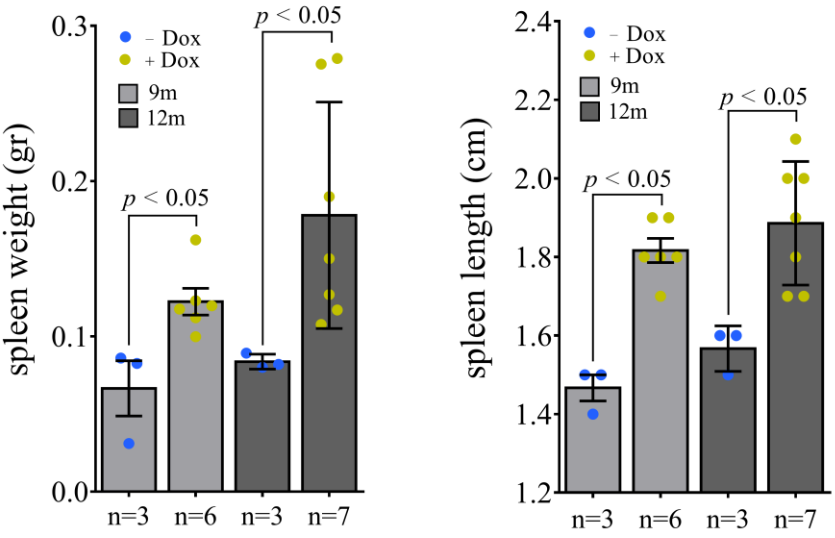

Supplementary Figure S3.
Presence of T cells in the Lymphomas of Rosa Vav1 mice. Sections of lung, liver, pancreas, and spleen from Rosa Vav1 mice treated with Dox, 12-months post transgene induction, were stained with anti-CD3 antibodies. Representative pictures are shown. Magnification at x10 and x20.

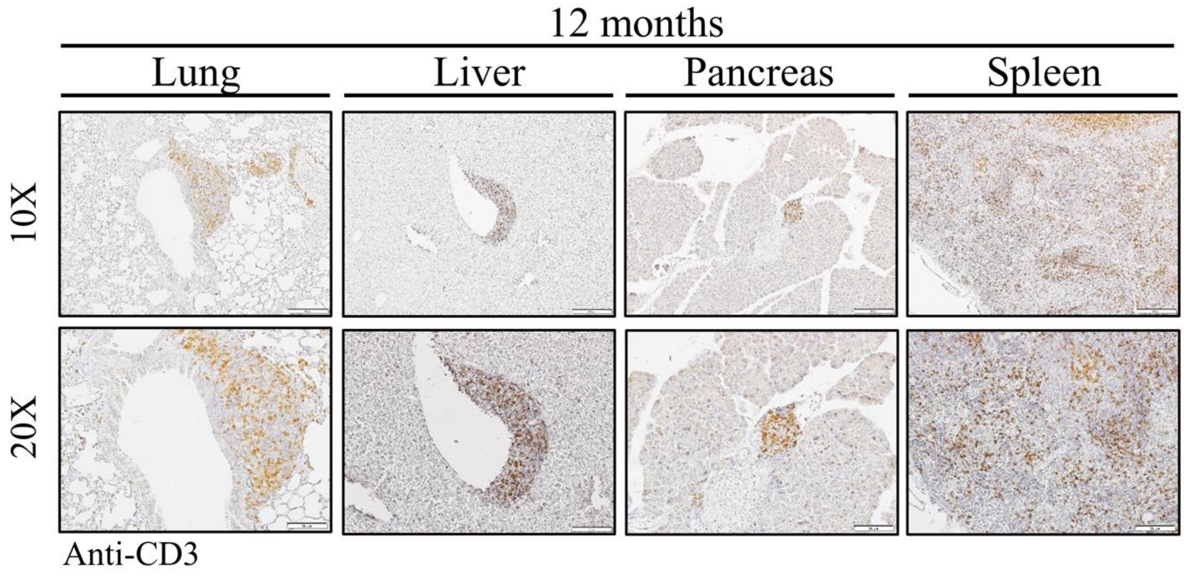

Supplementary Figure S4.
Vav1 activity as GEF for Rac in liver tissue from Rosa Vav1 mice. A. Lysates from livers from Rosa Vav1 mice either untreated with Dox (-, 1&2) or treated with Dox (+, 3-5), 12-months post transgene induction, were incubated with GST-PAK bacterial fusion proteins immobilized on glutathione sepharose beads (GST-PAK binding). Bound proteins were separated on SDS–PAGE and immunoblotted with anti-Rac1 mAbs. Expression of Rac1 in the various tissue lysates was determined by western blotting with anti-Rac mAbs as indicated (Tissue lysate) (upper panel). As a control for the validity of the assay, lysates of liver tissue (3 and 5) tested for GST-PAK pull down (upper panel) were either bound to GST only (lysate 5) or spiked with GTP-γ-S (lysate 3) and subjected to a Rac-GTP pulldown, as indicated (lower

panel). B. The relative ratios of Rac1-GTP/Rac1 was calculated from the blots shown in A. The mean intensity of the Western blots was quantified using ImageJ 1.49V software. SEM and significance between the treated (+Dox) and the non-treated (-Dox) analyzed by t-test are indicated.

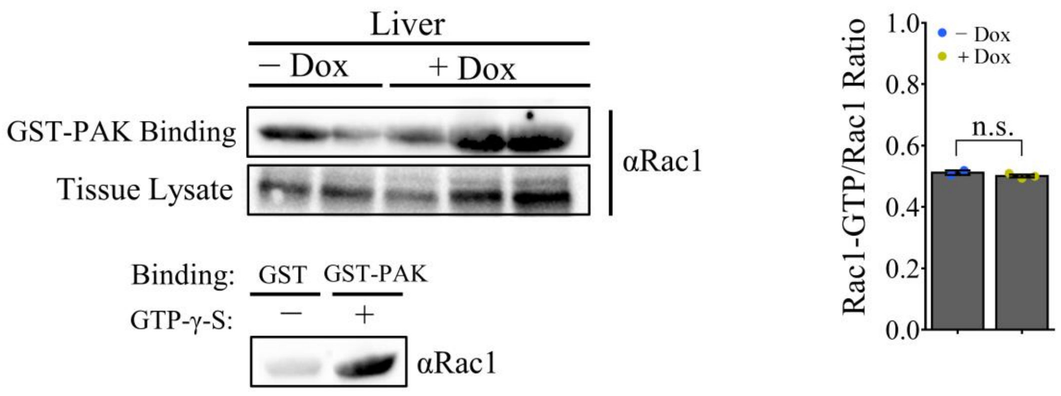

Supplementary Table S1: Primers used in this study

A. Primers for Mouse Genotyping

**Transgene**	**Primers**	
TetO-Vav1	Forward	Vav1 F: 5'-AGGGTGACATCATCAAGATCCTTAACAA-3'
	Reverse	GFP-R: 5'-TGCAGATGAACTTCAGGGTCAGCTT-3'
Rosa-rtTA		5'-AAAGTCGCTCTGAGTTGTTAT-3'
		5'-GCGAAGAGTTTGTCCTCAATT-3'
		5'-GGAGCGGGAGAAATGGATATG-3'

B. Primers for Real-Time PCR

**Transgene**	**Primers**	
GFP	Forward	5'-AGAACGGCATCAAGGTGAAC-3'
	Reverse	5'-TGCTCAGGTAGTGGTTGTCG-3'
Mouse Vav1	Forward	5'-CCGGATCACAGAGAAGAA GG-3'
	Reverse	5'-TGATGGCTCTCCTCTCAG GT-3'
UBC	Forward	5'-CAGCCGTATATCTTCCCAGACT-3'
	Reverse	5'-CTCAGAGGGATGCCAGTAATCTA-3'
HPRT	Forward	5'-GTTAAGCAGTACAGCCCCAAA-3'

	Reverse	5'-AGGGCATATCCAACAACAAACTT-3'

Supplementary Table S2. ***List of antibodies used in this study***

**Antibody**	**Application**	**Company**
Anti-Vav1	IHC	Cell signaling (#2505)
Anti-Vav1	WB	Millipore (#05-219)
Anti-ERK	WB	Abcam (#Ab17942)
Anti-pERK	WB	Millipore (#AW39R)
Anti-GFP	IHC, IF, WB	Abcam (#Ab6673)
Anti Rac1-GTP	WB	New East Biosciences (#26903)
Anti Rac1	WB	Cytoskeleton, inc. (ARC03)
Anti-B220	IHC	R&D MAB1217
Anti-CD3	IHC	Bio-Rad (MCA1477)
Anti-CSF-1R	IHC	Cell signaling (#3152S)
Anti-CSF-1	IHC, IF	ABGENT (AJ1466a)
Goat Anti-Mouse IgG (Alexa Fluor® 594)	IF	Invitrogen (Cat # A-21125)
